# The relationship between clinical symptoms of oral lichen planus and quality of life related to oral health

**DOI:** 10.1186/s12903-024-04326-2

**Published:** 2024-05-13

**Authors:** Maryam Alsadat Hashemipour, Sahab Sheikhhoseini, Zahra Afshari, Amir Reza Gandjalikhan Nassab

**Affiliations:** 1 Kerman Social Determinants On Oral Health Research Center, Kerman University of Medical Sciences, Kerman, Iran; 2 Department of Oral Medicine, School of Dentistry, Kerman University of Medical Sciences, Kerman, Iran; 3 Dentist. Member of Kerman Social Determinants On Oral Health Research Center, Kerman University of Medical Sciences, Kerman, Iran; 4 General Dentist, Private Practice, Shiraz, Iran; 5 Department of Otorinology, University of Medical Sciences, Isfahan, Iran

**Keywords:** OHIP-14, Oral lichen planus, Quality of life

## Abstract

**Introduction:**

Oral Lichen Planus (OLP) is a chronic and relatively common mucocutaneous disease that often affects the oral mucosa. Although, OLP is generally not life-threatening, its consequences can significantly impact the quality of life in physical, psychological, and social aspects. Therefore, the aim of this research is to investigate the relationship between clinical symptoms of OLP and oral health-related quality of life in patients using the OHIP-14 (Oral Health Impact Profile-14) questionnaire.

**Materials and methods:**

This descriptive-analytical study has a cross-sectional design, with case–control comparison. In this study, 56 individuals were examined as cases, and 68 individuals were included as controls. After recording demographic characteristics and clinical features by reviewing patients' records, the OHIP-14 questionnaire including clinical severity of lesions assessed using the Thongprasom scoring system, and pain assessed by the Visual Analog Scale (VAS) were completed. The ADD (Additive) and SC (Simple Count) methods were used for scoring, and data analysis was performed using the T-test, Mann–Whitney U test, Chi-Square, Spearman's Correlation Coefficient, and SPSS 24.

**Results:**

Nearly all patients (50 individuals, 89.3%) reported having pain, although the average pain intensity was mostly mild. This disease has affected the quality of life in 82% of the patients (46 individuals). The patient group, in comparison to the control group, significantly expressed a lower quality of life in terms of functional limitations and physical disability. There was a statistically significant positive correlation between clinical symptoms of OLP, gender, location (palate), and clinical presentation type (erosive, reticular, and bullous) of OLP lesions with OHIP-14 scores, although the number or bilaterality of lesions and patient age did not have any significant correlation with pain or OHIP scores.

**Conclusion:**

It appears that certain aspects of oral health-related quality of life decrease in patients with OLP, and that of the OLP patient group is significantly lower in terms of functional limitations and physical disability compared to the control group. Additionally, there was a significant correlation between clinical symptoms of OLP and pain as well as OHIP scores.

## Introduction

Lichen planus (LP) is a chronic and relatively common mucocutaneous disease that often affects the oral mucosa. The exact cause of the disease is yet to be discovered; however existing evidence suggests the involvement of immunologic processes in the etiology of the lesions. The disease is more common in women and middle-aged people, with an estimated prevalence ranging from 1% to 2.2% [1].

In the oral mucosa, LP typically presents as white lesions, often with erosions. The most common clinical pattern is the reticular form [1–4]. The most frequently affected oral sites are the buccal mucosa and, subsequently, the tongue and gingiva. Furthermore, the reticular, erosive, and bullous clinical patterns are common [5, 6].

The prevalence of LP lesions and other epidemiological parameters reported in various studies vary significantly. One major reason for these variations is the differences in research methodologies, study populations, sampling techniques, and sample sizes. Many studies have been conducted in dental clinics and hospitals [2–4], and population-based studies are limited [5, 6]. Given that many cases of oral LP are asymptomatic, and the possibility that these studies may not encompass all cases, this issue is raised. Moreover, the presence of lichenoid lesions as a broad spectrum of lesions with similar clinical and sometimes histological features can complicate the accurate diagnosis of LP [7].

Numerous clinical indices have been developed and refined based on clinical experience for the classification of oral LP [5]. Clinical features includes size, color, and location-based distribution [5]. The common clinical signs and symptoms of oral LP range from a burning sensation to severe chronic pain [4]. The measurement of pain associated with oral LP has been widely used in clinical practice and research [8–11].

Despite the availability of pain rating scales, none are capable of comprehensive assessment of the multidimensional aspects of pain [12]. Oral lichen planus is generally not life-threatening. However, the consequences of oral lichen planus can lead to the worsening of the quality of life in physical, psychological, and social dimensions. Effects such as difficulty eating certain foods, which can lead to weight loss or malnutrition in severe cases, have been reported. Dietary satisfaction is at risk and can impact happiness and social abilities [13, 14].

Furthermore, speech problems that may result from dry mouth have also been reported [15]. Additionally, the presence of an ulcerative lesion can restrict the performance of daily oral hygiene activities [16]. In terms of sleep disturbances, patients with oral lichen planus have more sleep disorders compared to healthy individuals [17]. It appears that sleep deprivation can amplify pain signals and increasing pain sensitivity [18].

Some studies have shown that patients with oral lichen planus experience higher levels of stress and anxiety compared to healthy individuals [19, 20]. Dissatisfaction with the appearance of oral lichen planus lesions on the lips, including whiteness, keratotic plaques, atrophic erythematous areas, or ulcers, as well as hyperpigmented coffee-colored or black areas following inflammation, has been reported [21–25], and this potentially affects the quality of life of patients due to its impact on aesthetics.

In relation to the social burden, it was investigated the aspects of OLP, including social cost, work loss or school absence, are related to the economy [26]. Lastly, it was revealed that the impact of OLP could cause the avoidance of social interactions, such as social gatherings or eating-out parties [13].

The concept of Oral Health-Related Quality of Life** (**OHRQoL) had been developed and introduced into all fields of dentistry, including oral medicine [24]. For clinicians, the application of OHRQoL revealed the importance of understanding the disease from the patient’s perspectives. Moreover, the goal of OLP treatment should focus, not only on healing the lesion and reducing pain, but also improving OHRQoL. Taking these factors into considerations, we believe that using merely clinical indicators is not sufficient, and the added value of subjective patients’ symptoms and OHRQoL in the research studies are anticipated [5, 24]. A number of previous studies have examined OHRQoL in OLP patients [27–38]. Most studies were conducted with the cross-sectional design. Various patient-based outcomes were used, for example, pain, self-perceived oral health, oral health satisfaction, as well as OHRQoL indices. Among the studies that applied the OHRQoL index, the Oral Health Impact Profile index (OHIP) was most frequently used [11, 28, 31–34, 36, 39]. The OHIP consists of 49 or 14 items (short form) covering a wide range of patient’s symptoms and problems of oral functioning. Therefore, the OIDP measures the changes in daily life performances which are considered as the ultimate oral impacts caused by various perceived symptoms [40].

Therefore, the aim of the present study was to assess OHRQoL of OLP patients using the OHIP index. Furthermore, the associations of OHRQoL and pain perception with OLP clinical characteristics in terms of localization, type, number and severity, according to Thongprasom sign scoring system were examined.

## Method

This study employed a descriptive-analytical and cross-sectional design with a case–control approach. Inclusion criteria for the case group included patients aged 18 or older who had been clinically and histopathologically diagnosed with oral lichen planus and confimed diagnosis. The clinical diagnosis of lichen planus was based on white lesion with Wickham’s striae in the forms reticular (fine white striae cross each other in the lesion), popular, erythematousor atrophic (areas of erythematous lesion surrounded by reticular components), ulcerative or erosive, plaque and Bullous. Also, the three classical histological feature of oral lichen planus what were put forward first by Dubreuill in 1906 and Shklar was used in this study (liquefaction degeneration of basal layer, overlying keratinization, lymphocytic infiltrate within the connective tissue that is dense and resembles a band) [24].

Additionally, the onset of their lesions should have occurred less than 3 years ago. On the other hand, exclusion criteria for the case group consisted of patients with other oral mucosal lesions, pregnant, smokers, and people with other oral mucosal changes and medical conditions which can have an additive role in the psychology of the patient and that could potentially affect their quality of life.

Furthermore, a total of 68 individuals with healthy oral mucosa were included as the control group. Inclusion criteria for the control group were participants aged 18 or older with no oral lesions or medical conditions such as diabetes that could affect their quality of life.

To conduct the study, patient records were reviewed, and demographic information, including gender, age, lesion type, time since the initial diagnosis of oral lichen planus, and clinical characteristics, were recorded. Additionally, phone contact was established with patients to assess pain severity and complete the OHIP-14 questionnaire.

A total of 56 individuals were examined in the case group and 68 individuals with healthy oral mucosa were included as the control group based on similar studies' sample sizes (z: 1.96, *p* = q = 0.5, d = 0.05).

The clinical severity of lesions was assessed using the Thongprasom scoring system [6], where scores ranged from 1 to 5, with 1 meaning only mild white lines, 2 meaning white lines with atrophic area < 1 square centimeter, 3 meaning white lines with atrophic area ≥ 1 square centimeter, 4 meaning white lines with erosive area < 1 square centimeter, and 5 meaning white lines with erosive area ≥ 1 square centimeter. In the case of multiple oral lichen planus lesions, the highest score among all lesions was recorded.

Regarding pain assessment, participants were asked to rate their current pain intensity related to oral lichen planus on a Visual Analog Scale (VAS), ranging from 0 to 10, where 0 indicated no pain, and 10 represented the worst imaginable pain. Pain scores were categorized into mild (0–3), moderate (4–7), and severe (8–10) [12].

The Oral Health Impact Profile (OHIP-14) questionnaire, which had a valid Persian version, was used to evaluate the quality of life of the patients [26]. This questionnaire comprised 14 items assessing various aspects of mental functioning and quality of life. It included seven subdomains: functional limitations, physical pain, psychological discomfort, physical disability, psychological disability, social disability, and handicap, with each subdomain containing two questions.

Two methods were employed to assess the responses: The Additive method and the Simple Count (SC) method. In the first method, the options of the questionnaire were scored as follows: 0 = never, 1 = rarely, 2 = sometimes, 3 = often, and 4 = always. The OHIP-14 score ranged from 0 to 56, with lower scores indicating better quality of life. Additionally, a "severity" measure was calculated to represent better mental perception. The severity scores were categorized into five groups: very low, low, moderate, severe, and very severe. In the SC method, options were scored as 0 for never and rarely, and 1 for sometimes, often, and always. This method was considered to account for the possibility that some individuals might not perceive the real difference between the questionnaire options. The OHIP-14 score ranged from 0 to 14 [27].

Data analysis was conducted using the T-test, the Mann–Whitney U test, the Chi-Square, Spearman's Correlation Coefficient, and SPSS Version 24. The significance level for data analysis was set at *P* < 0.05.

## Results

In this case–control study, 56 patients with histopathologically confirmed oral lichen planus and 68 healthy individuals, who had no complaints of oral mucosal diseases and had either accompanied patients or visited the School of Dentistry for routine dental examinations, were respectively enrolled as the case and control groups. The case group consisted of 36 females and 20 males, with a mean age of 48.2 ± 4.3 years, a minimum age of 39, and a maximum of 64 years. These two groups were matched in terms of age, gender, and oral health status (*P* = 0.12, 0.41, 0.23, respectively). Table 1 displays the demographic characteristics and oral health status of the participants.

**Table 1:** Demographic characteristics and oral health compliance status in 2 control and case groups

Variable		Case Group		Control Group		*P* Value
		No	%	No	%	
Gender	Male	20	35.7	24	35.29	0.41
	Female	36	64.28	44	64.7	
Mean Age		48.2 ± 8.3		43.9 ± 9.7		0.23
Age range	Minimum	39		35		0.21
	Maximum	64		60		
Marital Status	Married	45	80.35	47	69.11	0.08
	Single	11	19.64	21	30.88	
Education	≥ Diploma	31	55.35	38	55.88	0.06
	< Diploma	25	44.64	30	44.11	
Occupation	Employed	39	69.64	42	61.76	0.31
	Unemployed	17	30.35	26	38.23	
Monthly Income	≥ 100$	41	73.21	49	72.05	0.17
	< 100$	15	26.78	19	27.09	
Number Dental Visits in the Past Year	≥ 2 times	44	78.57	53	77.94	0.15
	< 2 times	12	21.42	15	22.05	
Toothbrushing	Yes	46	82.14	56	82.35	0.08
	No	10	17.85	12	17.64	
Dental Floss	Yes	24	42.85	20	29.41	0.05
	No	32	57.14	48	70.58	
Mouthwash	Yes	12	21.42	10	14.7	0.25
	No	44	78.57	58	85.29	
Oral Health Status	Good	10	17.85	14	20.58	0.12
	Average	31	55.35	42	61.76	
	Poor	15	26.78	12	17.64	

Twenty-two individuals (39.3%) among the participants had oral lichen planus lesions for one year, 18 of them (32.1%) between one to three years, and 16 of them (28.6%) had lesions for less than one year. Almost all patients (50 individuals—89.3%) complained of pain; however, the average pain intensity was primarily mild (34 individuals—60.7%), followed by moderate (14 individuals—25%), and the rest (8 individuals—14.3%) reported severe pain. The mean pain score was 3.1 ± 0.9.

Considering the clinical features of oral lichen planus, the commonly affected mucosal sites were buccal mucosa (78.2%), followed by gingiva (62.5%), tongue and lips (17.6%), palate (16.1%), and floor of the mouth (3.9%). Equal to 46.2% (23 individuals) had a reticular and popular type of oral lichen planus, 22% (13 individuals) had a combination of reticular, atrophic, and erosive types, 14.3% (8 individuals) had atrophic, 10.7% (6 individuals) had ulcerative, and finally, 10.7% (6 individuals) had bullous lesions. Regarding the distribution of oral lichen planus lesions, approximately 46.3% were bilateral, and the rest involved more than two sites.

The impact of oral lichen planus on the quality of life is presented in Table 2. About 82% (46 individuals) of patients stated that oral lichen planus have affected their quality of life. The total OHIP-14 score was 10.12 ± 18.15 in the case group and 8.71 ± 15.11 in the control group, with no statistically notable difference between the two groups (*P* = 0.05). The mean and standard deviation of OHIP-14 subgroups in each of the case and control groups using two evaluation methods are shown in Tables 2, 3 and 4. As observed, the case group had a greater functional limitation compared to the control group (*P* = 0.03). Also, using the SC evaluation method, the patient group reported significantly lower quality of life in terms of functional limitation and physical disability (*P* = 0.01, 0.02, respectively). There was a statistically noticeable difference between the mean total OHIP-14 score and its subgroups among genders (men more than women, *P* = 0.01). There was no significant difference between the mean total OHIP-14 score and its subgroups concerning age (*P* = 0.09).

**Table 2:** Mean and Standard Deviation under Subgroups of the OHIP-14 Questionnaire Using Two Scoring Methods in 2 Control and Case Groups

Subgroups of OHIP 14	Score ADD (Mean ± Standard Deviation)			Score SC (Mean ± Standard Deviation)		
	Case Group	Control Group	*P* Value	Case Group	Control Group	*P* Value
Functional Limitation	2.18 ± 1.9	1.36 ± 1.2	0.03*	1.41 ± 0.75	0.54 ± 0.55	0.02*
Physical Pain	2.21 ± 1.2	2.18 ± 1.1	0.08	1.31 ± 0.55	1.01 ± 0.32	0.84
Psychological Discomfort	3.32 ± 1.5	2.21 ± 1.4	0.14	1.01 ± 0.31	0.41 ± 0.46	0.03*
Physical Disability	3.25 ± 1.1	2.05 ± 1.2	0.06	0.86 ± 0.68	0.37 ± 0.35	0.01*
Psychological Disability	2.71 ± 1.7	2.04 ± 1.3	0.12	1.31 ± 0.82	1.24 ± 0.62	0.51
Social Disability	2.65 ± 1.9	2.15 ± 1.1	0.09	0.85 ± 0.76	0.34 ± 0.52	0.35
Handicap	2.42 ± 1.7	1.21 ± 1.2	0.28	1.02 ± 0.72	0.61 ± 0.21	0.07
Total OHIP-14	10.12 ± 18.15	8.71 ± 15.11	0.05	6.22 ± 3.21	5.21 ± 3.61	0.21

***P*** < 0.05 is significant

This study demonstrated a positive statistical correlation between clinical symptoms of oral lichen planus, pain, and the OHIP-14 questionnaire score. With an increase in the Thongprasom Sign Score, the OHIP-14 score increased. Pain in patients with oral lichen planus was associated with clinical severity, and a significant relationship was observed in this regard Table 3.

**Table 3:** Investigation of the Relationship and Distribution of the Severity of OHIP-14 Questionnaire Scores and the Level of Pain in Oral Lichen Planus Lesions Based on the Thongprasom Sign Score

Thongprasom Sign Score	No	%	Score Severity OHIP14	CC* P value	Mean ± SD of VAS	CC* P value
1	26	41.1	Very Low	rs = 0.421*** *P* = 0.001	1.22 ± 2.31	rs = 0.241*** *P* = 0.01
2	12	21.4	Low		1.42 ± 2.61	
3	9	16	Moderate		1.76 ± 2.91	
4	7	12.5	Severe		2.45 ± 3.56	
5	5	9	Very Severe		2.02 ± 3.79	
Total	56	100	Moderate		3.1 ± 0.9	

*Rs* spearman’s correlation coefficient

^*^Correlation Coefficient

^**^correlation is significant at the 0.05 level (2-tailed)

^***^ correlation is significant at the 0.001 level (2-tailed)

^#^ *p* < 0.05 (Mann–Whitney U test) compare to one-step lower clinical severity scores

The location and clinical manifestation type of oral lichen planus lesions were related to the OHIP-14 questionnaire score. The study showed that oral lichen planus in the palate significantly affected the OHIP-14 score, leading to a significant increase in the score. Patients with ulcerative, erosive, and bullous types of oral lichen planus reported remarkably higher pain levels compared to other types. Although the number of lesions did not have any correlation with pain and questionnaire score. Table 4

**Table 4:** Investigation of the relationship between erosive oral lichen planus, reticular oral lichen planus, the number of oral lichen planus lesions, and the severity of OHIP-14 questionnaire scores and pain level

Variable		No	%	Pain Severity	*P* value	Mean ± SD of VAS	*P* value
Palate	Yes	9	16.1	Moderate	*0.02	2.21 ± 2.34	0.72
	No	47	83.9	Severe		0.56 ± 3.12	
Erosive, Atrophic, and Bullous	Yes	23	35.7	Moderate	*0.001	2.17 ± 2.04	*0.001
	No	33	64.3	Severe		2.07 ± 3.54	
One Site		12	21.4	Moderate	0.45	3.15 ± 4.12	0.26
Two Sites		26	46.6	Moderate		2.11 ± 1.78	
Three Sites		7	12.5	Severe		2.17 ± 2.81	
Four Sites		7	12.5	Moderate		2.76 ± 3.45	

^*^ *P* < 0.05. Mann–Whitney U test

## Discussion

Lichen planus is a relatively common chronic skin disease that often affects the oral mucosa. Patients with oral lichen planus suffer from symptoms that affect their daily life in various fields. Although the etiology of oral lichen planus is not known, the role of mental disorders, especially stress, anxiety and depression, in the pathogenesis of the disease is discussed [23–25].

Chronic diseases of the oral mucosa can definitely affect the quality of life. Therefore, several studies have investigated the quality of life related to oral health of patients with oral symptoms [28–31]. Patients with erosive lichen planus suffer from symptoms that affect their daily life in various fields. There are different tools and questionnaires for evaluating the quality of life related to oral health. These tools are used to complete clinical evaluations and strengthen the relationship between patients and physician, also patients can have a better understanding of the consequences of oral diseases in their daily life and their impact on quality of life [31].

OHIP-14 is a questionnaire that was first used by Slade in 1997 to evaluate the quality of life related to oral health. This questionnaire examines 7 aspects of the quality of life related to oral health, including functional limitation, physical pain, mental discomfort, physical disability, mental disability, social disability and disability [28, 32]. LOCKER model shows the effect of oral conditions on these 7 aspects of quality of life. Based on this model, the first level of factors affecting the quality of life related to oral health are functional limitations, physical pain and mental discomfort. At the next level, there are many factors that cause more problems in people's lives, which include physical, mental, and social disability, and finally, people may feel disabled in life due to oral diseases, which includes the last level of this model [31].

In this case–control study, 56 patients with confirmed lichen planus were considered as the case group and 68 healthy individuals who had visited Kerman Dental School for routine dental examinations \ without any muco-oral disease, were included in the study under the title of control group. The case group included 36 women and 20 men. The average age was 48.2 ± 4.3 years and they were at least 39 and at most 64 years old.

Twenty-two (39.3%) of the participants had oral lichen planus lesions for 1–5 years. 18 people (32.1%) had the lesion for more than 5 years and 16 people (28.6%) for less than 1 year. Almost all patients (50 people—89.3%) complained of pain. However, the average intensity of pain was mostly mild (34 people-60.7%), followed by moderate (14 people-25%) and the rest (8 people-14.3%) severe. The average pain score was 3.1 ± 0.9.

In Khalili and Shojaei's study [32], the mean age of the patients was 42 ± 14.2, and the patients ranged in age from 6 to 73 years. Silverman et al. [33, 34] in 2 studies reported the mean age as 52 years (22–80 years) and 54 years (21–82 years).

Equal to 46.2% (23 people) of the patients had reticular and popular type of lichen planus. 22% (13 persons) were a combination of reticular, atrophic and erosive types, 14.3% (8 persons) were atrophic, 10.7% (6 persons) were ulcers and finally 10.7% (6 persons) were bullous. According to the number of oral lichen distribution, about 46.3% were bilateral and the rest involved more than two places.

In Khalili and Shojaei's study [32], it was reported that the frequencies of female and male patients are 49.6% and 50.4%, respectively. The studies by Silverman and colleagues [33, 34] revealed that 65 to 67% of patients are women, and Vincent and colleagues reported this rate to be 76% [35]. Silverman et al. [33] found that the frequency of reticular lesions as 34% and the type of injury as 59.9%, and in another study, the frequency of reticular lesions was 28.5% and the type of injury was 71.58% [34]. In Vincent et al.'s research work [35], the frequencies of reticular, atrophic and ulcreated lesions were 24.3%, 33.6% and 41.9%, respectively.

Due to the fact that reticular lesions are not biopsied in most cases, the results of this study do not reflect the actual distribution of the disease in the population. In the mentioned studies, the amount of atrophic and injured type is more than the reticular type, and the reason for this is the examination of patients referred to diagnostic and treatment centers. It is obvious that because the reticular type has no pain and clinical symptoms, the referrals of affected people and even their awareness of the lesion are less than other types of diseases.

According to the clinical features of oral lichen planus, the three most common sites were buccal mucosa (78.2%), followed by gums (62.5%), tongue and lips (17.6%), palate (16.1%) and floor of the mouth (3.9%).

In the study by Khalili and Shojaei [32], the most common sites of involvement were the mucous membrane of the cheek and gums, followed by the tongue, and in 67% of cases, involvement was seen in only one anatomical site. The common conflict is consistent with all the researches that have been done before [33–35]. In the studies by Khalili and Shojaei [32] and Myers et al. [36], lesions have been presented in several areas of the mouth in most cases.

Based on the results of this research, the quality of life related to the oral health of the patient group was lower than that of the healthy group, and the patients with oral lichen planus expressed significantly more functional limitations and physical disability than the healthy group. Functional limitation in many patients was due to their dissatisfaction with the change in the taste of the mouth, and their physical disability was mostly due to dissatisfaction with the type of food they were eating. This finding is in accordance with the research of Tebelnejad et al. [27]. Based on the investigation by Lopez-Jornet et al. [28], who examined the quality of life related to oral health in patients with oral lichen planus in Spain the patients' quality of life was slightly lower than the control group and the patients' quality of life was reported to be lower in terms of mental disability, social disability and disability.

The difference between the findings in the study by López-Jornet et al. [28] and those obtained in the present work can be related to the different population under study and the sample size.

Ashshi et al.'s research [37] showed that oral lichen planus has significantly poorer quality of life in Chronic Oral Mucosal Disease Questionnaire-26 (COMDQ-26) and Oral Potential Malignant Disorder QoL Questionnaire (OPMDQoL) compared to dysplasia. In addition, patients with oral lichen planus aged 40 to 64 years were independently associated with higher COMD-26 scores compared to older patients (> 65 years).

The present investigation depicted that there is a significant relationship between the type of ulcerative, atrophic and bullous lesion and the presence of a lesion in the palate and increased pain intensity.

The increase in pain and irritation in the oral mucosa of patients with oral lichen planus can be a reason for the effect on the functional and physical aspects of the patients' quality of life and on the effect of lichen disease, which has also been found in the study of Hegarty and colleagues [30]. The oral plan emphasizes the quality of life and its physical, social and psychological aspects.

In the research of Saberi et al. [38] on patients with erosive/ulcerative OLP, there was a significant relationship between oral pain and the total score of COMDQ as well as its physical, social and emotional domains.

In this research, the total score of OHIP-14 in the case group was 18.15 ± 10.12 and in the control group was 15.11 ± 8.71, without any statistically significant relationship between the two groups, such that the case group had more functional limitations than the control group. Also, by using the SC evaluation method, the patient group expressed a significantly lower quality of life compared to the healthy group in terms of aspects of functional limitation and physical disability.

The study of Daume et al. [39] showed that the average score of OHIP-14 in the case group is 13.54 and there is a significant difference between the two groups. There was a significant difference in the areas of physical pain, mental discomfort, physical disability and social disability. Physical pain score and eating restriction score were significantly different between clinical forms.

Although in the present study it seems that oral lichen planus disease has caused the quality of life of people to decrease, "according to the decrease in the quality of life in the first and second levels of the LOCKER model, it has not led to the third level of disability in the LOCKER model, which is confirmed by the research by Tebelnejad et al. [27].

The quality of life related to oral health of patients referred to oral diseases England, and also people with oral diseases and functional limitation, physical pain and discomfort was studied by Llewellyn and colleagues [31] and Slade [40]. They faced more mental problems than the general population. Although these diseases have caused a lower quality of life according to the first level of the LOCKER model, they have not caused disability.

Osipoff et al. [41] showed that erosive lichen planus is not significantly related to the increase in pain intensity, which is consistent with the findings of Gonzalez-Moles et al. [42]. Research by Suliman et al. [43] and Hegarty et al. [44] reported more severe pain and quality of life problems in patients with erosive lichen planus.

Our findings showed that pain intensity doesn’t have any relation with bilateral lesions. These results are in accordance with other findings [13, 27, 45–47]. However, Osipoff et al. [41] found that lichen planus is the most painful lesion, which is not in agree with our results.

The results of Wiriyakijja et al.'s study [48], which is consistent with previous researchs [49, 50], showed that patients with ulcerative lichen planus experienced a greater impact on quality of life than those with other clinical types. Also, patients with ulcerative lichen planus reported significant levels of oral discomfort when eating certain foods, performing health care, more concerns about medication use, and more psychosocial burden. This finding is consistent with a previous study, which showed the change and avoidance of diet in patients with lichen planus regardless of the presence of ulcerative/erosive lesions [51]. Therefore, it seems that regardless of the clinical type, the presence of lichen planus have a negative effect on various types of patient activities and all oral symptoms such as pain [52, 50].

Vilar-Villanueva et al. [53] found a higher OHIP-14 score for patients with atrophic/ulcerative lichen planus compared to patients with reticular lichen planus. Karbach et al. [54] reported similar findings. However, Parlatescu et al. [55] did not find a significant difference between asymptomatic and symptomatic lichen planus patients. They attributed this observation to the small number of clinical subtypes of lichen planus, but Wiriyakijjia et al. observed a poor quality of life score in ulcerative lichen planus patients compared to keratotic lichen planus patients [56].

As discussed above, these preliminary results of association analyses from current investigation were subject to certain limitations. First, our cross-sectional data would not allow for evaluating the effects of OLP treatment on OHRQoL. The data were mostly derived from follow-up patients, while 15.2% of patients were newly diagnosed who never previously been treated. For recall patients, information on OLP treatment was not available. Treatment experience in terms of type and duration of treatment might affect patient’s quality of life. Two previous longitudinal studies following OLPpatients after treatments reported significantly improved clinical signs, as well as OHRQoL [33, 34].

Therefore, further longitudinal study to assess overtime change of OIDP intensity, taking into account previous or ongoing treatment, would be required for better understanding on the impacts of OLP treatment on patients’ quality of life. Second, some of the previous studies performed multivariate analysis where confounding factors were taken into account [28, 35]. The others limitation was non-cooperation of a number of patients and Incomplete number of files.

However, this study applied only univariate analyses due to a relatively small sample size. The small sample size led to the third limitation on the generalization of our findings to OLP patients, particularly for reticular OLP as discussed earlier. Therefore, future study with larger sample size is required in order to corroborate the present study’s findings.

The current study demonstrated that nearly all patients had oral impacts affecting their daily activities. The impacts were frequently related to eating, cleaning the oral cavity and emotional stability. There were significant associations between OLP clinical signs and OHRQoL. However, some increasing clinical scores did not correspond with the increase of OHRQoL. Therefore, using only an OLP sign scoring index or other clinical indicators might fail to acknowledge patient’s perceptions. The results supported the application of OHRQoL assessment to complement OLP clinical measures.

## Conclusion

It seems that some aspects of the quality of life related to oral health are reduced in patients with lichen planus. The quality of life related to oral health in the group of patients with lichen planus is significantly lower in terms of functional limitations and physical disability was more than the control group. There was also a significant relationship between the clinical symptoms of lichen planus and pain.

### Limitation

Non-cooperation of a number of patients.

Incomplete number of files.

Otherwise the limitation of this finding was relatively small numbers of patient with soft palate involvement.

Our cross-sectional data would not allow for evaluating the effects of OLP treatment on OHRQoL.

## Data Availability

The datasets used and/or analyzed during the current study available from the corresponding author on reasonable request.
